# Indole Derivative Interacts with Estrogen Receptor Beta and Inhibits Human Ovarian Cancer Cell Growth

**DOI:** 10.3390/molecules25194438

**Published:** 2020-09-27

**Authors:** Laura Verardi, Jessica Fiori, Vincenza Andrisano, Alessandra Locatelli, Rita Morigi, Marina Naldi, Carlo Bertucci, Elena Strocchi, Carla Boga, Gabriele Micheletti, Natalia Calonghi

**Affiliations:** 1 Department of Pharmacy and Biotechnology, University of Bologna, 40121 Bologna, Italy; laura.verardi88@gmail.com (L.V.); alessandra.locatelli@unibo.it (A.L.); rita.morigi@unibo.it (R.M.); marina.naldi@unibo.it (M.N.); carlo.bertucci@unibo.it (C.B.); 2 Department of Chemistry ‘G. Ciamician’, University of Bologna, Via Selmi, 2, 40126 Bologna, Italy; jessica.fiori@unibo.it; 3 Department for Life Quality Studies, University of Bologna, Corso D’Augusto 237, 47921 Rimini, Italy; vincenza.andrisano@unibo.it; 4 Centre for Applied Biomedical Research–CRBA, University of Bologna, St. Orsola Hospital, 40126 Bologna, Italy; 5 Department of Industrial Chemistry ‘Toso Montanari’, University of Bologna, Viale Del Risorgimento, 4, 40136 Bologna, Italy; elena.strocchi@unibo.it (E.S.); gabriele.micheletti3@unibo.it (G.M.)

**Keywords:** indole derivative, ovarian cancer, estrogen receptor beta, histones, LC/ESI/MS, molecular docking

## Abstract

Ovarian cancer remains the leading cause of mortality among gynecological tumors. Estrogen receptor beta (ERβ) expression has been suggested to act as a tumor suppressor in epithelial ovarian cancer by reducing both tumor growth and metastasis. ERβ expression abnormalities represent a critical step in the development and progression of ovarian cancer: for these reasons, its re-expression by genetic engineering, as well as the use of targeted ERβ therapies, still constitute an important therapeutic approach. 3-{[2-chloro-1-(4-chlorobenzyl)-5-methoxy-6-methyl-1*H*-indol-3-yl]methylene}-5-hydroxy-6-methyl-1,3-dihydro-2*H*-indol-2-one, referred to here as compound **3**, has been shown to have cytostatic as well cytotoxic effects on various hormone-dependent cancer cell lines. However, the mechanism of its anti-carcinogenic activity is not well understood. Here, we offer a possible explanation of such an effect in the human ovarian cancer cell line IGROV1. Chromatin binding protein assay and liquid chromatography mass spectrometry were exploited to localize and quantify compound **3** in cells. Molecular docking was used to prove compound **3** binding to ERβ. Mass spectrometry-based approaches were used to analyze histone post-translational modifications. Finally, gene expression analyses revealed a set of genes regulated by the ERβ/3 complex, namely CCND1, MYC, CDKN2A, and ESR2, providing possible molecular mechanisms that underline the observed antiproliferative effects.

## 1. Introduction

Ovarian cancer is the second most common cancer in women over the age of 40, particularly in developed countries [1]. If compared to all types of cancers, it is the eleventh most common type in women, the fifth leading cause of cancer related death in women, and the most fatal gynecologic cancer [2].

Estrogens, as the main female sex steroids, have important functions in the regulation of cellular processes such as growth and differentiation in human ovary and endometrium. Their effects are mediated by two receptors, namely estrogen receptor alpha (ERα) and estrogen receptor beta (ERβ), which belong to the nuclear receptor superfamily and act as ligand-activated transcription factors [3]. ERβ is known to act as an ERα antagonist in specific settings, and their balance is important for their action [4,5,6]. Overall, ERα is thought to promote the expression of genes involved in cell survival and proliferation, thus determining tumor growth and progression, while the natural function of ERβ is thought to be antiproliferative and proapoptotic, and ERβ is therefore described as a tumor suppressor. Indeed, it is known that the antiproliferative effect of ERβ is accompanied by downregulation in the expression of CYCLINE D1 (CCND1), a cell cycle regulator overexpressed in epithelial, ovarian, and breast cancers [7,8,9,10]. Several ERβ isoforms have been reported thus far; in the normal ovary, the levels of ERβ are high and predominant over ERα, being ERβ1, β2, and β5 the most represented ones [11]. Therefore, ERβ is the only fully functional isoform that is able to form homodimers or heterodimers with ERα, whereas ERβ2 and β5 do not form homodimers and have no innate activities of their own, but may modulate estrogen action when dimerized with ERβ or ERα [12]. Unfortunately, studies on the functional roles of ERβ2 and β5 in ovarian cancers are limited. In some papers, the authors have demonstrated ERβ5-induced cell migration, invasion, and proliferation, while ERβ2 was shown to affect ovarian cell migration and invasion, but not proliferation [13,14]. Moreover, ERβ2 has been reported to correlate with chemoresistance [15].

Other authors have reported that in ovarian epithelial tumors there is a loss of ERβ expression and a relationship of the ERβ/ERα ratio, compared to normal ovarian tissue [6,7,8,9,10,11,12,13,14,15,16]. Furthermore, a complete loss of ERβ was observed in the metastases of ovarian tumors, while low expression levels were still measurable in the presence of primary tumors [17]. A positive correlation of ERβ expression with survival has been demonstrated in patients with ovarian cancer and animal models [18,19]. If ERβ expression abnormalities represent a critical step in the development and progression of several cancers including ovarian cancer, its re-expression by genetic engineering, as well as the use of targeted ERβ therapies, may constitute important new therapeutic approaches. 

**Figure 1:**
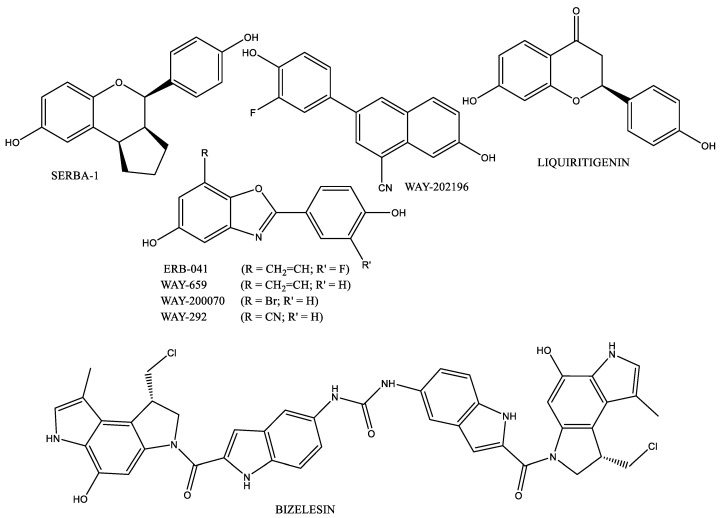
Non-steroidal selective ERβ agonists belonging to different classes of organic compounds.

Among them, an important role is played by the development of efficient ERβ agonists. Recently, estrogenic activities of non-steroidal selective ERβ agonists such as SERBA-1, 20], WAY-202196 [20], and Liquiritigenin [21] have been reported (Figure 1). Other ERβ agonists are ERB-041 [20], WAY200070 [22], WAY-29 [23], and WAY-659 [23] (Figure 1). 

**Scheme 1:**
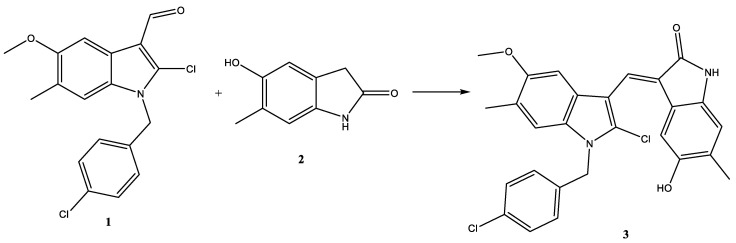
Synthesis of compound **3** [25].

Despite the well-known activity conferred by the presence of the indole scaffold in drugs and pharmaceuticals, including anticancer agents, benzoxazole is the main heterocyclic moiety of many ERβ agonists. However, some indole moieties are in the structure of Bizelesin, a drug used in clinical trials of treatment of solid tumors, including ovary cancer [24]. In the past, we reported the synthesis and a first screening of the biological activity of a small library of Knoevenagel adducts, which were synthesized by reacting oxindoles (**2**) with indole aldehydes (**1**) properly substituted [25]. Among them, 3-{[2-chloro-1-(4-chlorobenzyl)-5-methoxy-6-methyl-1*H*-indol-3-yl]methylene}-5-hydroxy-6-methyl-1,3-dihydro-2*H*-indol-2-one (**3**) (Scheme 1) emerged as a lead compound owing to its significant growth inhibition of cancer cells. Actually, in a primary screening, compound **3** was subjected to the Developmental Therapeutics Program (DTP) at the National Cancer Institute (Bethesda, MD) where it came out to exert a cytostatic but not cytotoxic activity, restricted to leukemia, brain, ovarian, kidney, prostate, and breast cancer cell lines [25]. Compared to other compounds found to be active on ovarian cancer cell lines, compound **3** caused the accumulation of IGROV1 cells in the G0/G1 phase, not in the G2/M phase. Moreover, compound **3** showed no significant effect on tubulin polymerization, indicating that this compound does not interfere with microtubule dynamics, further differentiating it from other leads (i.e., **3g** and **3f** [25]) that arrest cells in mitosis, confirmed by the condensation of tubulin (marker of cytoskeleton reorganization). These promising results on compound **3** and the growing interest in the development of valid ERβ agonists motivated us to investigate more deeply its molecular mechanism of action as a possible ERβ agonist. Interestingly, from an experimental point of view, this compound is fluorescent and can be monitored by fluorescence-based methods (excitation at 488 nm and emission at 530 nm). 

In this paper, we report the results of the studies on the impact of this molecule on the proliferation of ovarian cancer cell line IGROV1, by confirming the effect of compound **3** on cell growth; investigating the expression of key cell cycle regulators, ERβ colocalization, and changes in the histones post-translational modifications pattern; and performing molecular docking.

## 2. Results

### 2.1. Biological Activity

#### 2.1.1. Compound **3** Inhibits IGROV1 Ovarian Cancer Cells Proliferation and Progression in the Cell Cycle

**Figure 2:**
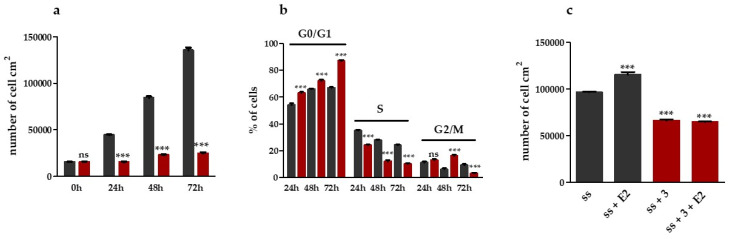
Compound **3** inhibits IGROV1 cells growth: (**a**) IGROV1 cells viability after treatment with **3** (red) is compared with untreated cells (gray); (**b**) IGROV1 cells were treated with compound **3** (red) and the isolated nuclei stained with PI were analyzed by flow cytometry; and (**c**) E2 induces proliferation in serum starved (ss) but not in compound **3** treated IGROV1. Cells were serum starved or treated with 5 µM compound **3** for 24 h. Each group was then stimulated with 1 nM E2 and cell growth measured. Statistical analysis was carried out using Prism GraphPad *** *p* < 0.001. n.s., not significant.

IGROV1 cells were treated with compound **3** (5 µM, corresponding to the concentration that induces 50% of growth inhibition) for 72 h and the cell number determined by the Trypan Blue dye exclusion method. As depicted in Figure 2a, compound **3** drastically inhibits the proliferation of ovarian cancer cells. IGROV1 cells were treated with compound **3** for 24, 48, and 72 h and analyzed by flow cytometry after DNA staining. Untreated cells progress through the cell cycle; in contrast, compound **3** treated IGROV1 cells accumulate in the G0/G1 phase in a time-dependent manner, with a nearly complete synchronization (87%) after 72 h of treatment. The flow cytometric analyses along with the relative phase percentages are shown in Figure 2b. The set of experiments was designed to address whether cells withdrawn from the cell cycle by compound **3** could reenter it and proliferate in response to the administration of an exogenous hormone, 17β-estradiol (E2). As shown in Figure 2c, E2 treatment leads to an increase in proliferation in serum-starved cells (ss), but it does not exert any effect in compound **3** treated cells.

**Figure 3:**
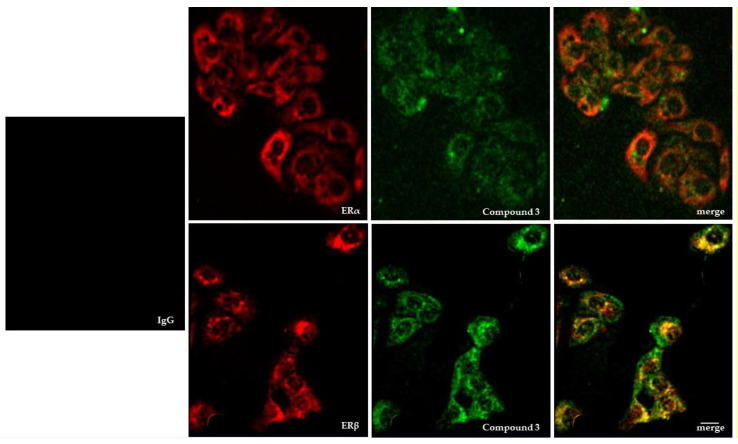
ERβ/**3** complex in IGROV1 cells. IGROV1 cells were treated with 5 μM **3** for 6 h, and then fixed and immunostained for ERα or ERβ (red) and compound **3** (green). IgG, negative control. Yellow pixels represent ERβ/**3** colocalization, indicating the formation of an ERβ/**3** complex. Bar = 20 µm.

ERα or ERβ interaction with compound **3** was investigated by confocal microscopy. Both ERs were visualized by fluorescent specific antibodies (red fluorescence) and compound **3** using its inherent fluorescence (green). Figure 3 shows how ERα and compound **3** are diffused in cellular compartments of IGROV1, but the merging indicates that they do not colocalize. On the contrary, ERβ and compound **3** colocalize mostly in the nuclear compartment. Unfortunately, a triple staining to highlight the nucleus with dyes such as DAPI and or Hoechst is not possible because the excitation spectrum of compound **3** partially overlaps with those of the nuclear dyes. For this reason, different techniques were exploited to assess the nuclear localization of the compound **3** and its interaction with the ERβ, thus confirming the interpretation of the results obtained by confocal microscopy.

#### 2.1.2. Compound **3**: Nuclear Localization, ERβ on Chromatin Colocalization, and Molecular Docking

**Figure 4:**
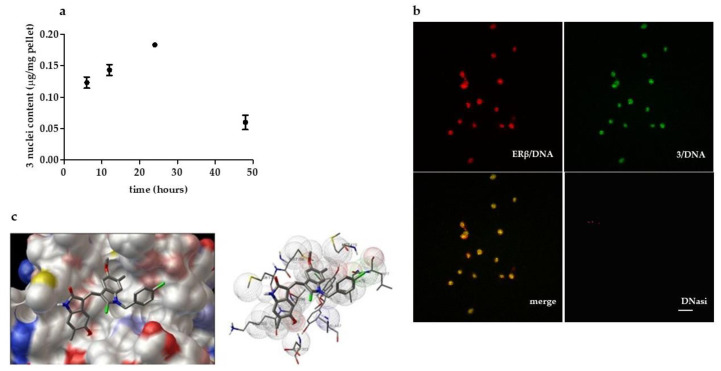
Nuclear localization, ERβ on chromatin colocalization, and molecular docking: (**a**) Nuclear content profile of compound **3** at different incubation times (*n* = 3). The results are obtained by analyzing the sample in SIM modality on the ESI generated ions at *m/z* 493 and the quantitation results from the total of *cis* and *trans* content. (**b**) ERβ/**3** complex binds chromatin. Yellow pixels represent ERβ/**3** colocalization. In the negative control, lysis was followed by DNase I treatment. Bar = 20 µm. (**c**) The images show the lowest energy conformation of compound **3** into the ERβ binding site: (left) the best docking binding pose of compound **3** is shown into the pocket binding site of ERβ; and (right) the amino acid residues of the ERβ binding site within 5 Å from compound **3** are displayed.

The LC-ESI-MS was validated for the nuclear localization and quantitative determination of compound **3**. The concentration–time profile of compound **3** in the IGROV1 nuclear fraction is shown in Figure 4a. Nuclear lipids from IGROV1 cells exposed for 6, 12, 24, or 48 h to compound **3** were extracted, and LC-MS analysis performed as described in the Supplementary Materials (Page *S2*). Compound **3** nuclear concentration (expressed as μg/mg pellet) was found to increase from 6 to 24 h and decrease at a concentration under the quantitation limit after 48 h.

Next, chromatin binding of ERβ and compound **3** was assessed. IGROV1 cells were treated with compound **3** for 6 h and then lysed by CSK + buffer to remove soluble proteins, leaving chromatin and chromatin-bound proteins (CBP) intact (Figure 4b). ERβ/**3** complex colocalizes on chromatin, and both ERβ and compound **3** staining are absent in DNase treated cells, demonstrating the dependence of specific signals on intact chromatin (Figure 4b).

In an attempt to explain these data at a molecular level, the interaction of compound **3** with ERβ was investigated by a computational approach. We analyzed the location and orientation of the evaluated compound and the interactions into the binding site of the selected Erβ. The predicted binding free energy (BE) was used as the criterion for ranking. The conformation with the lowest ranking docked binding energy (BE) was considered to be the “best” docking result. Docking on the interaction between the indole derivative **3** and ERβ was performed; virtual constants of inhibition (*K*i) at micromolar concentration were measured. Compound **3** docks and nicely fits into the active site of the ERβ, as indicated by the binding free energy [ΔG] reported (BE: −7.11 kcal/mol) and calculated KI (Constant of inhibition) equal to 6.19 µM. The analysis of compound **3** binding pose inside the ERβ active site shows that it can form a hydrogen bond between the –OH group of an indole moiety with THR299. Compound **3** is stabilized into the ERβ active site through interactions between the 5-Å surrounding polar MET295, MET296, and MET479 and the methoxy group of the indole moiety; between LYS300 and ASP303 residues and dihydro-indole-one moiety; and between aromatic LEU476, LEU477, and VAL487 and Cl-ethyl-benzene of compound **3**. The best binding pose of **3** into the ERβ active binding site is shown in Figure 4c.

#### 2.1.3. Mass Spectrometry-Based Assessment of Histone Acetylation

**Figure 5:**
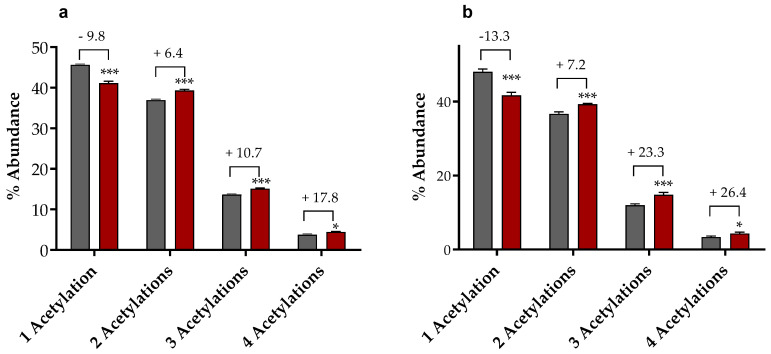
Compound **3** induces an increase of H4 acetylation degree. The histograms report the relative abundance of histone H4 isoforms grouped on the basis of their acetylation status obtained from the analysis of the deconvoluted ESI mass spectrum. Histones were extracted from IGROV1 cells after treatment with **3** (red) or from untreated cells (gray) for: 6 h (**a**); or 24 h (**b**). Above the bar, the percent difference between the control and compound **3** is reported. * *p* < 0.05, *** *p* < 0.001.

To assess if the treatment with **3** involves changes in the histones post-translational modifications (PTMs) pattern, mass spectrometry-based analyses of histones extracted from IGROV1 were carried out on control cells and after treatment with compound **3** (see Page S3); to this purpose, both top-down and bottom-up approaches were exploited as previously described [26,27]. In particular, the liquid chromatography–mass spectrometry (LC-MS) analysis performed on isolated histones allowed the identification and quantification of the most abundant PTMs. This highly informative approach showed that treatment with compound **3** induces a significant increment in the H4 acetylation degree while it does not affect the PTM profile of other histone proteins (data not shown). The global increase of the H4 acetylation degree translates, after treatment with compound **3**, into the increment of the relative abundance of the forms carrying more than one acetylation (from 2 to 4) with a concomitant decrease of the monoacetylated form (Figure 5). The increment in the acetylation degree was also found to be dependent on the incubation time of compound **3** in IGROV1 cells and it is therefore sharper after 24 h of treatment. 

MALDI-TOF analysis of the H4 peptides, obtained after the endoproteinase Arg-C digestion (Supplementary Materials: S4,S5, confirmed that the increment of the acetylation degree concerns the N-terminal tail of the protein (Figure S2).

#### 2.1.4. Compound **3** Induces Gene and Protein Expression Modulations

**Figure 6:**
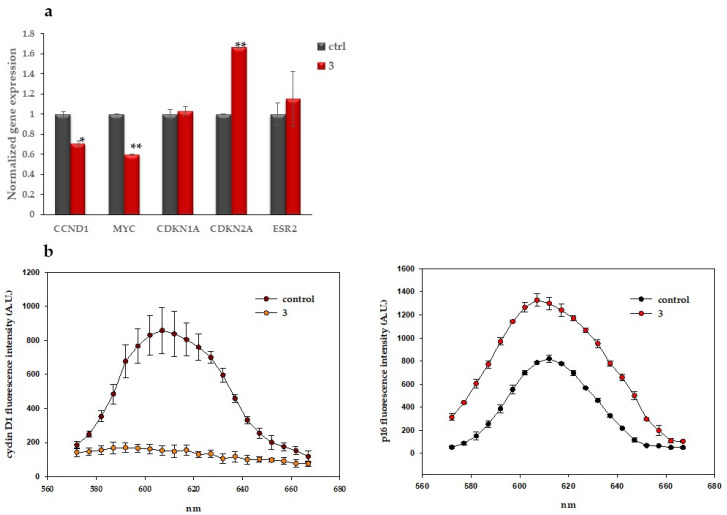
Compound **3** modifies gene and protein expression: (**a**) gene expression for CCND1, MYC, CDKN1A, CDKN2A, and ESR2 analyzed by RT-PCR after 6 h of treatment; and (**b**) fluorescence spectra of cyclin D1 (left) and p16 (right) under 568 nm laser excitation. Data shown are the result of five separate experiments. * *p* < 0.05, ** *p* < 0.01.

**Figure 7:**
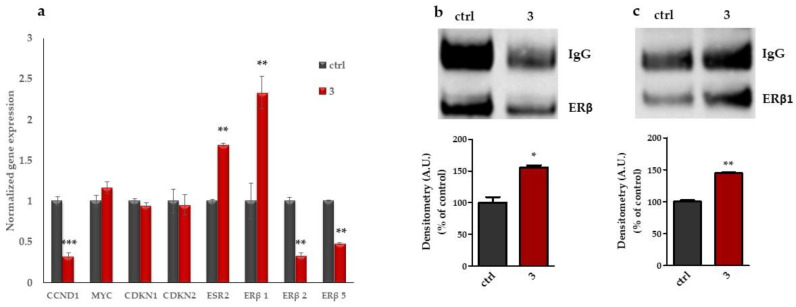
Compound **3** modifies ESR2 isoform expression in IGROV1: (**a**) gene expression for CCND1, MYC, CDKN1A, CDKN2A, ESR2, *ERβ1*, *β2,* and *β5* analyzed by RT-PCR after 24 h of treatment; and (**b**,**c**) immunoprecipitation and Western blot analysis of ERβ and β1 (top). Cell lysates from control and IGROV1 treated with **3** for 48 h were immunoprecipitated with anti ERβ or β1 antibodies; and (bottom) relative quantification of ERβ and β1. Densitometry arbitrary units (A.U.) were normalized by IgG. Results are given as means ± SD of three independent experiments and are compared to controls, taken as 100%. * *p* < 0.05, ** *p* < 0.01.

To investigate the observed cytostatic effect of compound **3**, transcription levels of CCND1, c-MYC (MYC), P21 (CDKN1A), P16 (CDKN2A), and ERβ (ESR2) genes, which control cell proliferation, were analyzed by Real-Time Polymerase Chain Reaction (RT-PCR). The analysis was performed on cDNA of control and compound **3**-treated cells, and the ΔΔC_T_ method was used with G3PDH as the housekeeping gene (a list of primers for quantitative RT-PCR is shown in Table S1). Relative transcription levels after 6 or 24 h treatment, expressed as means of fold changes, are reported in Figure 6a and Figure 7a, respectively.

At 6 h of treatment, compound **3** administration does not affect CDKN1A and ESR2 transcription, while it significantly upregulates CDKN2A and simultaneously downregulates CCND1 and MYC. Cyclin D1, c-myc, and p16 protein expressions were analyzed by spectral confocal microscopy in control and 24-h treated cells. Compound **3** administration determines a drastic decrease in cyclin D1 (Figure 6b) and similar results were obtained for c-myc (data not shown), while it significantly increases p16 expression (Figure 6b), causing an arrest of cell proliferation.

At 24 h of treatment, the most significant modifications of gene expression are represented by a downregulation of CCND1 and an upregulation of ESR2, while there are no differences in the expression of MYC, CDKN1, and CDKN2. Interestingly, the increase in ESR2 is due to an overexpression of the ERβ1 isoform alone, while the β2 and β5 isoforms decrease significantly (Figure 7a). Figure 7b shows how the levels of ERβ have increased, and Figure 7c shows that the increase in protein contents of this receptor is supported by the ERβ1 isoform, while those of cyclin D1 remain decreased (data not shown).

## 3. Discussion

In a previous work, the antiproliferative effect induced by compound **3** in different ovarian cancer cell lines has been described [25]. Here, we continued the studies on the effects of compound **3** using, as a model, an ovarian cancer line, IGROV1, which expresses both ERα and ERβ receptors.

Compound **3** administered to IGROV1 decreases cell growth by 66% already 24 h after treatment and by 82% after 72 h (Figure 2a). Such a decrease is due to a time-dependent arrest in the G0/G1 phase of the cell cycle, with a nearly complete synchronization (87%) after 72 h of treatment (Figure 2b).

We then explored the effect on IGROV1 growth of the non-subtype-selective, natural hormone E2, both alone and associated to compound **3**. In the presence of both ER subtypes, compound **3** has a dominant inhibitory effect on cell growth, antagonizing the growth stimulatory effect of ERα (Figure 2c). The colocalization analysis reveals that **3** forms a complex only with ERβ (Figure 3). Moreover, the binding of the ERβ/**3** complex to DNA demonstrates that the effects on cell proliferation induced by compound **3** are due to its interaction with ERβ (Figure 4b). Its molecular interaction with ERβ was also confirmed by using a computational approach (Figure 4c).

ERβ levels and/or the ERβ /ERα ratio decrease along with ovarian carcinogenesis, indicating that the loss of ERβ expression may be involved in carcinogenesis [16,18,28,29].

We then focused on exploring the effect of compound **3** on the histone acetylation and gene expression in IGROV1. At 6 h of treatment, compound **3** induces an increase of acetylated H4 isoforms (Figure 5) and a downregulation of MYC and CCND1 genes (Figure 6a), acting as an ERβ agonist, in accordance with other findings on other agonists [30,31].

Moreover, we did not find significant changes in the expression of CDKN1A and ESR2 genes, but, interestingly, a significant upregulation of CDKN2A gene (Figure 6a) and the corresponding protein product p16 (Figure 6b) was found. CDKN2A is located on chromosome 9p21 and plays an important role in cell cycle regulation by slowing cells progression from G1 to S phase [32,33]. It has become clear that the expression of CDKN2A is reduced by DNA methylation [18,34,35]. In addition, CDKN2A inactivation upregulates RB, stimulating the cyclin-dependent kinases (CDKs) and the RB pathway, which leads to malfunction of cell proliferation and apoptosis, thereby further facilitating carcinogenesis [36]. Indeed, several types of cancer, including ovarian cancer, exhibit a methylation phenotype of CDKN2A [37,38,39]. Interestingly, other authors have suggested that aberrant methylation of CDKN2A promoter may be essential to the initiation of ovarian cancer and in distinguishing malignant from healthy ovarian tissues. Besides, CDKN2A promoter methylation is a potential predictive factor for poor prognosis in ovarian cancer [40].

At 24 h of treatment, we repeated the analysis on gene expression and epigenetic modifications. LC-MS analysis of the purified histone H4 showed an increase of acetylation. The state of histone acetylation is closely associated with chromatin remodeling and gene regulation. Indeed, most significant modifications of gene expression are represented by a downregulation of CCND1 and an upregulation of ESR2 genes (Figure 7a). Interestingly, the increase in ESR2 is due to an overexpression of the ERβ1 isoform alone, while the β2 and β5 isoforms decrease significantly. This result is particularly important in ovarian tumors. In fact, it has been reported that a low level of ERβ1 may contribute to ovarian cancer metastasis, while β5 and β2 induce cell migration, invasion, and proliferation [14] Other authors have found that overexpression of ERβ1 represses in vitro cell migration and invasion in ovarian cancer cells [41,42], as well as reduces tumor formation in sites of metastasis in vivo [18].

## 4. Materials and Methods 

### 4.1. Cell Culture and Chemicals

The human ovarian cancer cell line IGROV1 was kindly provided by Istituto Nazionale Tumori Milano, Italy. Cells were maintained in RPMI 1640 medium (Labtek Eurobio, Milan, Italy), supplemented with 10% FBS (Euroclone, Milan, Italy) and 2 mM L-glutamine (Sigma-Aldrich, St. Louis, MO, USA) at 37 °C and 5% CO_2_. 

3-{[2-Chloro-1-(4-chlorobenzyl)-5-methoxy-6-methyl-1*H*-indol-3-yl]methylene}-5-hydroxy-6-methyl-1,3-dihydro-2*H*-indol-2-one (**3**) was dissolved in dimethyl sulfoxide (DMSO) (Sigma-Aldrich, St. Louis, MO, USA) at a concentration of 20 mM. The drug was diluted in fresh medium at the final concentration of 5 µM, according to Andreani et al. [25]. 17-beta Estradiol (E2) (Sigma-Aldrich, S.Louis, MO, USA) was dissolved in DMSO at a concentration of 20 mM and diluted in fresh medium at a work concentration of 1 nM.

#### 4.1.1. Proliferation Assays and Cell Cycle Analysis

IGROV1 cells were plated in 6-well dishes; after 48 h, the medium was removed and fresh medium containing the drugs added. Control cells received the corresponding volume of DMSO. The number of viable cells was determined by trypan blue exclusion.

For E2 response assay, IGROV1 cells were plated in 6-well dishes at equal density and grown in 10% FCS for 48 h. Cells were serum-starved for 24 h and then stimulated with E2 1 nM, with or without 5 μM compound **3**, for an additional 24 h. Untreated cells were used as controls. Viable cells were counted by using Trypan blue exclusion dye method.

For cell cycle assay IGROV1 cells were treated for 24, 48, and 72 h with compound **3**, detached with 0.11% trypsin (Sigma-Aldrich, St. Louis, MO, USA)/0.02% ethylenediaminetetraacetic acid (EDTA) (Sigma-Aldrich, St. Louis, MO, USA), washed in PBS, and centrifuged. Flow cytometric analysis was performed as previously described. [43]

#### 4.1.2. Confocal Microscopy

IGROV1 cells were grown on glass coverslips and exposed to compound **3** 5 µM for 24 or 48 h. Cells were fixed, permeabilized, and incubated with primary antibodies against ERα, ERβ, (Santa Cruz Biotechnology, Dallas, TX, USA), cyclin D1 (Millipore, Billerica, MA, USA), or p16 (Santa Cruz Biotechnology, Dallas, TX, USA) overnight at 4 °C, followed by appropriate Alexa 568-conjugated secondary antibody. IGROV1 cells were pre-exposed to negative control (IgG isotype) and then samples were fixed and stained with the secondary and fluorescent antibody (Alexa fluor-568). Specimens were embedded in Mowiol and analyzed in confocal microscopy by using a Nikon Plan Apo 60X oil objective.

For spectral imaging, fluorescence was detected in spectral mode in the interval 572–667 nm at 5-nm resolution. At least 60 cells were measured and the signal corrected for background fluorescence by subtracting the fluorescence detected from the extracellular region in the same spectral image. Images were captured with a Nikon C1s confocal laser-scanning microscope (Nikon, Tokyo, Japan).

For the chromatin binding assay, IGROV1 cells were grown on glass coverslips and exposed to compound **3** for 6 h. To visualize chromatin-bound proteins, cells were processed as described previously [44,45]. Briefly, cells were lysed in CSK + buffer for 30 min in ice, and then fixed and incubated with primary antibody ERβ (Santa Cruz Biotechnology, Dallas, TX) overnight at 4 °C. Subsequently, samples were washed with 1% Bovine Serum Albumin (BSA)/PBS and stained with appropriate Alexa 568-conjugated secondary antibody for 1 h, at room temperature. To ensure that proteins remaining after in situ extraction were bound to intact chromatin, cells were treated with 200 U/mL DNase I (Promega, Madison, WI, USA) for 30 min at 37 °C after being lysed in CSK + buffer. Specimens were embedded in Mowiol (Hoechst, Frankfurt, Germany) and multiple images acquired by using sequential laser excitations at 488 and 568 nm to reduce spectral bleed-through artifacts. The images were collected by using a Nikon C1s confocal laser-scanning microscope, equipped with a Nikon PlanApo 60X, 1.4-NA oil immersion lens.

#### 4.1.3. Immunoprecipitation and Western Blot Analysis 

IGROV1 cells were lysed for 1 h in lysis buffer according to Calonghi et al. [46]. Cell lysates were centrifuged, supernatant collected, and protein concentration determined by using the Bio-Rad protein assay method (Bio-Rad, Hercules, CA, USA). For immunoprecipitation, an aliquot of 500 µg of proteins was incubated overnight at 4 °C with 1 µg of anti-ERβ or anti-ERβ1 (Abcam, Cambridge, UK) antibody. Then, Protein A–Sepharose was added to each sample according to Parolin et al. [45]. The proteins were resolved by SDS–PAGE and immunoblotted with anti-ERβ or anti-ERβ1 antibody. Detection of immunoreactive bands was performed with a secondary antibody conjugated with horseradish peroxidase and developed with an enhanced chemiluminescence (ECL) system (GE Healthcare, Milan, Italy). Densitometry analysis was performed by Fluor-S Max MultiImager (Bio-Rad, Hercules, CA, USA), and relative quantification of both ERβs was done by using IgG signal as a control.

#### 4.1.4. LC-MS Based Assessment of 3 in IGROV1 Nuclei

An LC-ESI-MS based analytical method was developed and validated for the three quantitative determinations of IGROV1 nuclei. The experimental details are reported in the Supplementary Materials (Page S2).

#### 4.1.5. MS-Based Determination of Histones Acetylation Degree

The LC-MS investigation of IGROV1 histone PTMs was performed by applying the analytical method previously reported by Naldi et al. [27] and here summarized in the Supplementary Materials (Page S3). The histone H4 acetylation degree was also investigated by MALDI-TOF analysis following a previously optimized analytical method [47] as described in the Supplementary Materials (Page S4).

#### 4.1.6. Molecular Docking

Molecular docking was employed to verify and understand the binding mode of **3**–ESR2 interaction. The ESR2 structure was retrieved from the Protein Data Bank (PDB ID: 2QTU). 

Binding modes and binding affinities of the evaluated compound within the binding site of the selected estrogen receptor ESR2 were calculated using the Autogrid 4.0 and Autodock 4.2 programs [48]

The docking calculations were then performed using the Lamarckian genetic algorithm (LGA) for ligand conformational searching to estimate the possible binding conformations of our indole derivative. During the docking process, a maximum of 150 different conformations was considered for compound **3**. The conformer with the lowest binding free energy was used for further analysis.

#### 4.1.7. Quantitative RT-PCR

IGROV1 cells were treated with compound **3** for 6 or 24 h. Total RNA was isolated by RNeasy Mini kit (Qiagen, Hilden, Germany) according to the manufacturer’s protocol. One microgram of RNA was reverse transcribed with RevertAid First Strand cDNA Synthesis Kit (Fermentas, Ontario, Canada) by using oligo(dT) primers. The cDNA was analyzed by RT-PCR, by employing the LightCycler FastStart DNA Master SYBR Green I Kit and the LightCycler 2.0 instrument (Roche Diagnostics, Manheim, Germany). Gene expression was quantified by the ΔΔC_T_ method using G3PDH as the housekeeping gene. The list of primers for quantitative RT-PCR are reported in Table S1. Amplicon specificity was verified by first-derivative melting curve analysis and agarose gel electrophoresis.

#### 4.1.8. Statistical Analysis

All data are expressed as mean ± SD (standard deviation). Student’s test was employed for the biological experiments, statistical differences were considered significant at a value of *p* < 0.05, and are reported as *p* < 0.05 (*), *p* < 0.01 (**), and *p* < 0.001 (***). ESI-QTOF and MALDI-TOF experiments were repeated at least three times, on three independent samples. ANOVA test was used for repeated measurement values. A *p* value below 0.05 was considered significant.

## 5. Conclusions

The results of this study strongly support the potential of compound **3** as an ERβ agonist for the treatment of ovarian cancer and provide evidence that it promotes tumor suppression pathways by modulating gene expression. The most important considerations that make this compound still interesting are as follows: (*i*) The effect that compound **3** has in common with other ERβ agonists is the downregulation of the CCND1 gene, while the effect that differentiates it is given by the upregulation of CDKN2A. A decrease in cyclin D1 associated with an increase in the p16 protein causes an arrest of proliferation in the G0/G1 phase of the cell cycle. As reported in the literature [49,50], cyclin D1 not only promotes the G1/S-phase transition by binding and activating Cdk4 and Cdk6, but it also plays a very important role in DNA damage repair mechanisms, especially in homologous recombination-directed repair (HDR). Cyclin D1 is recruited to damaged foci by BRCA2 and enhances RAD51 binding to BRCA2. Furthermore, cyclin D1 is essential for RAD51 gene upregulation in case of double-stranded break (DSB). Thus, the combined treatment of a drug leading to a depletion of cyclin D1 and a DNA damage inducer, such as irradiation, could be an interesting potential therapy to target cancers with a high rate of DNA repair, especially in the case of chemo- and radio-resistant tumors. (*ii*) Compound **3** induces a decrease in the *ERβ2–β5* isoforms’ gene expression. Unfortunately, studies on the functional roles of ERβ2 and β5 in cancers are limited, as are studies on their molecular mechanisms of action. However, recent papers show that ERβ5 and β2 play an important role in ovarian tumorigenesis by regulating cell migration, invasion, and proliferation, suggesting for them a role as potential prognostic markers and therapeutic targets [13,14].

## Supplementary Materials

Supplementary LC-ESI-MS and MALDI TOF material, list of primers for quantitative RT-PCR.
