# Supplementary material for: Indole Derivative Interacts with Estrogen Receptor Beta and Inhibits Human Ovarian Cancer Cell Growth
Source: Molecules. 2020 Sep 27;25(19):4438. doi: 10.3390/molecules25194438 (PMC7582771; doi:10.3390/molecules25194438)
Supplement: Supplementary file 1 [file molecules-25-04438-s001.pdf]

SUPPORTING INFORMATION

Indole Derivative Interacts with Estrogen Receptor Beta and Inhibits Human Ovarian Cancer Cell Growth

Laura Verardi [1], Jessica Fiori [2], Vincenza Andrisano [3], Alessandra Locatelli [1], Rita Morigi [1], Marina Naldi [1, 4], Carlo Bertucci [1], Elena Strocchi [5], Carla Boga [5, *], Gabriele Micheletti [5] and Natalia Calonghi [1, *]

[1] Department of Pharmacy and Biotechnology, University of Bologna, 40121 Bologna, Italy; laura.verardi88@gmail.com (L.V.), alessandra.locatelli@unibo.it (A.L.), rita.morigi@unibo.it (R.M.), marina.naldi@unibo.it (M.N.), carlo.bertucci@unibo.it (C.B.)

[2] Department of Chemistry 'G. Ciamician', University of Bologna, Via Selmi, 2, 40126 Bologna, Italy. jessica.fiori@unibo.it

[3] Department for Life Quality Studies, University of Bologna, Corso D'Augusto 237, 47921 Rimini, Italy vincenza.andrisano@unibo.it

[4] Centre for Applied Biomedical Research – CRBA, University of Bologna, St. Orsola Hospital, 40126 Bologna, Italy

[5] Department of Industrial Chemistry 'Toso Montanari', University of Bologna, Viale Del Risorgimento, 4 40136 Bologna, Italy; elena.strocchi@unibo.it (E.S.), gabriele.micheletti3@unibo.it (G.M.)

* Correspondence: carla.boga@unibo.it (C.B.); natalia.calonghi@unibo.it (N.C.); Tel.: +39-051-2093616 (C.B.); +39-051-2091231 (N.C.)

Table of content ***Page***

Assessment of nuclear content profile of 3.. S2

Nuclei extraction S2

LC-MS analysis.. S2

Results.. S3

Assessment of histone post-translational modifications.. S3

Histones treatment and extraction.. S3

LC-ESI-MS analysis.. S3

Assessment of the acetylation position on histone H4.. S4

Chromatographic purification of H4.. S4

H4 enzymatic digestion and MALDI-TOF analysis.. S4

Results MALDI-TOF analysis.. S5

RT-PCR S6

Table S1: List of primers for quantitative RT-PCR.. S6

References S6

Assessment of nuclear content profile of 3

Nuclei extraction

The nuclei of IGROV1 cells treated for 2, 6, 12, 24, or 48 h with **3** were extracted according to Amellem et al. [1] while nuclear lipids were extracted according to Folch, Lees, and Stanley [2]. The nuclei extracts (pellets) were added to 100 µL of a mixture of methanol:water:formic acid 50:50:0.1 (v/v), placed in an ultrasonic bath, centrifuged, and the supernatants were injected into the liquid chromatography–mass spectrometry (LC-MS) system.

LC-MS analysis

LC-MS analysis was carried out on a Jasco PU-1585 Liquid Chromatograph (Jasco Corporation, Tokyo, Japan) interfaced with a LCQ-Duo Mass Spectrometer (Thermo Finnigan, San Jose, CA). The mass spectrometer was equipped with an electrospray ionization (ESI) source and operated with an Ion Trap analyzer. ESI system was employed at 4.5 kV (positive polarity) spray voltage and heated capillary temperature of 220 °C. The sheath gas and the auxiliary gas (nitrogen) flow rates were set at 0.75 and 1.2 L/min, respectively. Electrospray ionization was optimized using **3** as the reference compound. The mass chromatograms were acquired in Total Ion Current (TIC) modality from 150 to 1000 *m*/*z*, and in Single Ion Monitoring (SIM) on the protonated **3** molecule at *m*/*z* 493, corresponding to the $[M + H]^+$ ion. Reverse phase chromatographic analyses were performed on a Waters Sunfire C18, 3.5 µm (100 × 2.1 mm I.D.) column, using a mobile phase consisting of methanol:water:formic acid 75:25:0.1 (*v:v:v*) at flow rate of 200 µL/min. The injector was a Rheodyne valve (model 7725i) with a 20-µL loop. Chromatographic separation allowed resolving **3** *cis* and *trans* isomers (Figure S1). The linearity of the ESI-IT-MS response to **3** concentration was verified by injecting freshly prepared standard solutions. A stock solution of **3** was prepared by dissolving the appropriate weight of the pure substance in methanol to obtain a final concentration of 0.2 mg/mL. Standard solutions for the calibration graph construction were prepared by diluting appropriate volumes of the stock with mobile phase to have final concentrations of 2.4, 4.8, 9.6, 15.0, and 24.0 µg/mL. Solutions of **3** were analyzed (SIM mode) by 20-µL loop injections, and the linearity of the response checked before each quantitative assay of **3**. The Limit of Detection (LOD) and the Limit of Quantitation (LOQ) were calculated.

Results

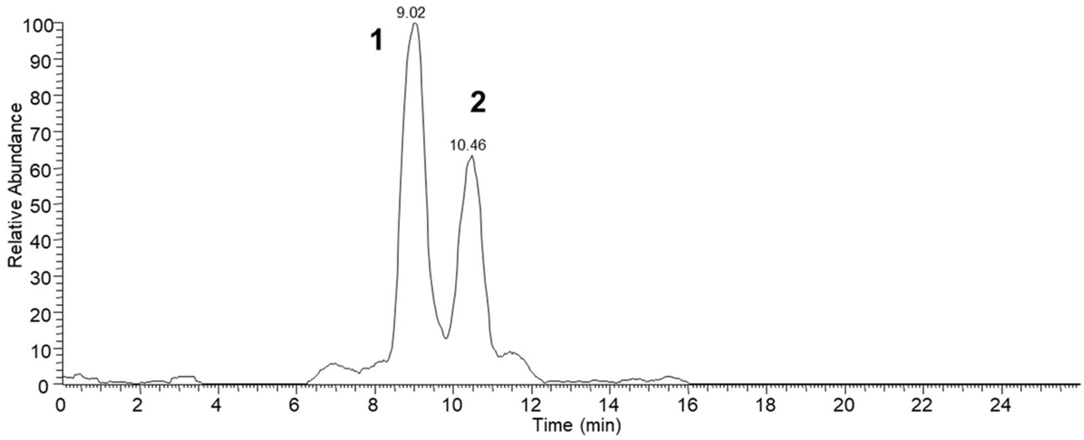

Figure S1. LC/MS chromatogram acquired in SIM modality on the protonated 3 molecule $[M + H]^+$ at *m*/*z* 493: Nuclei of 24 h treated cells, **3** *cis* and *trans* forms (Peaks 1 and 2, respectively).

Assessment of histone post-translational modifications

Histones treatment and extraction.

IGROV1 cells were cultured with or without 5 μM 3l for 6 h, and the histone fraction was immediately extracted. Cells were harvested using 0.11% trypsin and 0.02% EDTA, washed twice with 10 mM sodium butyrate (NaBU) in PBS, and nuclei were isolated according to Amellem et al. [1]. The nuclear pellet was suspended in 0.1 mL of ice-cold water using a Vortex mixer, and H_2SO_4 was added to the suspension to give a final concentration of 0.4 N. After incubation at 4 C for 1 h, the suspension was centrifuged for 5 min at 14,000 *g*, and the supernatant was taken and mixed with 1 mL of acetone. After overnight incubation at −20 °C, the coagulate material was collected by microcentrifugation and air-dried.

LC-ESI-MS analysis.

The histones PTMs were analyzed slightly adapting the analytical workflow previously reported [3] using an Agilent 1200 chromatography system (Agilent, Walbronn, Germany) interfaced with a quadrupole-time of flight (Q-TOF Micro, Micromass, Manchester, UK) hybrid mass analyzer equipped with a Z-spray electrospray (ESI) ion source. Briefly, the chromatographic separation of histones was performed on a C4 (Jupiter Phenomenex 5 mm, 2.0 ×150 mm i.d.) column, assembled with a precolumn SecurityGuardTM HPLC system consisting of a C4 guard cartridge (Phenomenex, 4.0 × 3.0 mm i.d.) inserted into its cartridge holder, using an elution gradient from A [water:heptafluorobutyric acid:formic acid (100:0.04:0.2) (*v:v:v*)]/B [ACN:heptafluorobutyric acid:formic acid (100:0.04:0.2) (*v:v:v*)] 65/35 v/v, to A/B 30/70 *v/v*, for 70 min, at a flow rate of 0.2 mL/min; the injection volume was 5 μL. The column was equilibrated with the mobile phase composition of the starting conditions for 10 min before the next injection. The ESI-Q-TOF source temperature was set at 100 °C, the capillary voltage at 3.5 kV and the cone voltage at 35 V. The scan time was set at 2.0 s and the interscan time at 0.1 s. The cone gas flow was set at 60 L/h and the desolvation gas at 500 L/h. Mass chromatograms were recorded in total ion current (TIC), within 800–1600 m/z. The histones baseline-subtracted spectrum (*m*/*z* 800–1600) was deconvoluted onto a true mass scale using the maximum entropy (MaxEnt1) software supplied with MassLynx 4.1 software. The abundance of single isoforms was calculated as the ratio between their fractional intensity and the sum of the intensities of all isoforms, expressed as percentage. Data were analyzed by Microsoft Excel software.

Assessment of the acetylation position on histone H4

Chromatographic purification of H4.

LC preparative separation of the histone H4 was performed using a Jasco PU-1585 liquid chromatograph (Jasco Corporation, Tokyo, Japan) with a Rheodyne 7281 injection valve (50-μL sample loop) interfaced with a UV detector (Jasco, UV 1575), fixed at 220 nm. The chromatographic method employed for the histone H4 purification is the same used for the LC-ESI-MS analysis. Repeated injections of histones sample were performed and histones H4 were eluted from the column, collected, and dried under vacuum. The purity of the isolated protein was checked under the same chromatographic conditions.

H4 enzymatic digestion and MALDI-TOF analysis.

Histone H4 was digested with endoproteinase Arg-C (Roche) in 20 mM ammonium bicarbonate buffer pH 8.0 at an enzyme ratio 1:50 at 37 °C overnight. Matrix assisted laser desorbtion ionization–time of flight (MALDI-TOF) analysis of the Arg-C digested was performed using a Voyager DE Pro (Applied Biosystems, Foster City, CA) equipped with a pulsed N_2 laser operating at 337 nm. Positive ion spectra were acquired in reflector mode over an m/z range of 500–4000 amu, using a 20,000-V accelerating voltage, a 14,900-V grid voltage, and an extraction delay time of 150 ns. The spectrum of each spot was obtained by averaging the results of 100 laser shots. Histones digest spectra were internally calibrated on 40–45 and 1–17 theoretical histone digest peptide masses that were sufficiently abundant in the spectra. The analysis was performed by spotting 1 μL of histone H4 digested sample mixed with an equal volume of matrix solution, consisting of CHCA (10 mg/mL) in water–acetonitrile–TFA (50:50:0.1, *v*/*v*/*v*), on the target plate.

Results MALDI-TOF analysis

To confirm that the increased level of the histone H4 acetylation, assessed by LC-MS analysis, was localized at the level of the N-term tail, the enzymatic digestion of the purified histone H4 followed by MALDI-TOF analysis was performed. Endoproteinase Arg-C was used as proteolytic enzyme to obtained peptides of length compatible with the MS detection. Among others, peptides 5–18 were identified as carrying different degrees of acetylation (from 0 to 5; Figure S1). The relative abundanceof these forms, derived from the analysis of sample treated or not with **3** for 6 and 24 h, confirmed the results achieved by the analysis on the intact protein. Indeed, **3** was found to induce an increment of the relative abundance of the forms carrying a higher number of acetylation (from 2 to 4) with a concomitant decrease of the less acetylated forms (Figure S1).

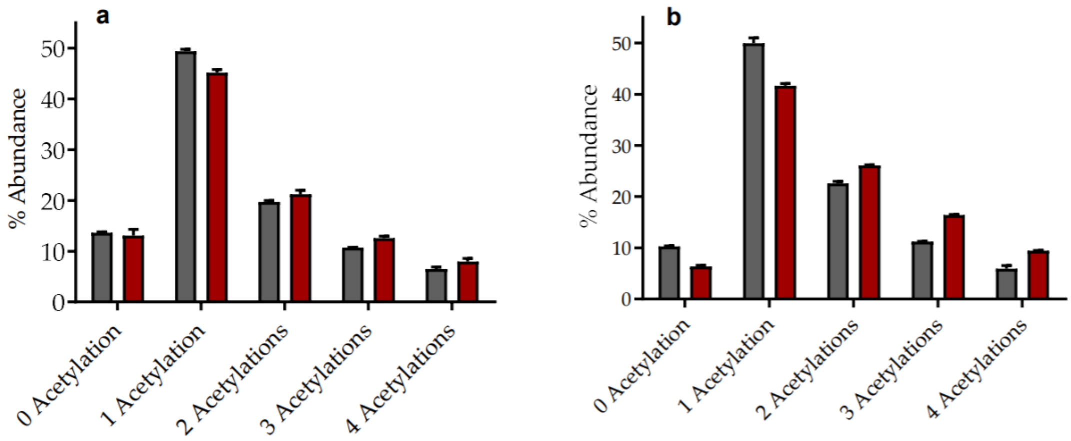

Figure S2. The histograms report the relative abundance of the peptide 5-18 derived from the MALDI-TOF analysis of the histone H4 digested with the endoproteinase Arg-C. Histones were extracted from IGROV1 cells after treatment with **3** (red) or from untreated cells (grey) for: 6 h (**a**); and 24 h (**b**).

RT-PCR

Table S1. List of primers for quantitative RT-PCR.

PRIMERS	SEQUENCE
G3PDH FW	ATTTGGTCGTATTGGGCGCC
G3PDH REV	ACGGTGCCATGGAATTTGCC
CDKN1 FW	CCTAAGAGTGCTGGGCATTTT
CDKN1 REV	TGAATTTCATAACCGCCTGTG
CCND1 FW	GCCAACTGGTGTTTGAAAGTA
CCND1 REV	TCCGGTGTGAAACATCTAAGA
CDKN2 FW	GGCTCCTCATTCCTCTTCCT
CDKN2 REV	TCAGGTAGCGCTTCGATTCT
MYC FW	TAG CTT CAC CAA CAG GAA CT
MYC REV	AGCTCGAATTTCTTCCAGAT
ESR2 FW	AGCGTGAGGATTCCCGTAGCTCT
ESR2 REV	GAGGCAAAATGTCTACTCTCCAGGC
ERβ1 FW	CCTGGCTAACCTCCTGATGC
ERβ1 REV	ACCCCGTGATGGAGGACTT
ERβ2 FW	GATCTTGTTCTGGACAGGGATG
ERβ2 REV	AGGCCTTTTCTGCCCTC
ERβ5 FW	CTTTGGTTTGGGTGATTG
ERβ5 REV	TTCCTTAACTTGCAGACACT

References

1. Amellem, O.; Stokke, T.; Sandvik, J.A.; Pettersen, E.O. The retinoblastoma gene product is reversibly dephosphorylated and bound in the nucleus in S and G2 phases during hypoxic stress. *Exp. Cell Res.* **1996**, *227*, 106–115.
2. Folch, J.; Lees, M.; Sloane Stanley, G.H. A Simple Method for the Isolation and Purification of Total Lipides From Animal Tissues. *J. Biol. Chem.* **1957**, *226*, 497-509.
3. Naldi, M.; Calonghi, N.; Masotti, L.; Parolin, C.; Valente, S.; Mai, A.; Andrisano, V. Histone post- translational modifications by HPLC-ESI-MS after HT29 cell treatment with histone deacetylase inhibitors., *Proteomics*, **2009**, *9*, 5437–5445.
